# Neuropeptide GPCRs in *C. elegans*

**DOI:** 10.3389/fendo.2012.00167

**Published:** 2012-12-21

**Authors:** Lotte Frooninckx, Liesbeth Van Rompay, Liesbet Temmerman, Elien Van Sinay, Isabel Beets, Tom Janssen, Steven J. Husson, Liliane Schoofs

**Affiliations:** ^1^Laboratory of Functional Genomics and Proteomics, Department of Biology, Katholieke Universiteit LeuvenLeuven, Belgium

**Keywords:** nematoda, *Caenorhabditis elegans*, G protein-coupled receptor, neuropeptidergic signaling, GPCR deorphanization

## Abstract

Like most organisms, the nematode *Caenorhabditis elegans* relies heavily on neuropeptidergic signaling. This tiny animal represents a suitable model system to study neuropeptidergic signaling networks with single cell resolution due to the availability of powerful molecular and genetic tools. The availability of the worm’s complete genome sequence allows researchers to browse through it, uncovering putative neuropeptides and their cognate G protein-coupled receptors (GPCRs). Many predictions have been made about the number of *C. elegans* neuropeptide GPCRs. In this review, we report the state of the art of both verified as well as predicted *C. elegans* neuropeptide GPCRs. The predicted neuropeptide GPCRs are incorporated into the receptor classification system based on their resemblance to orthologous GPCRs in insects and vertebrates. Appointing the natural ligand(s) to each predicted neuropeptide GPCR (receptor deorphanization) is a crucial step during characterization. The development of deorphanization strategies resulted in a significant increase in the knowledge of neuropeptidergic signaling in *C. elegans*. Complementary localization and functional studies demonstrate that neuropeptides and their GPCRs represent a rich potential source of behavioral variability in *C. elegans*. Here, we review all neuropeptidergic signaling pathways that so far have been functionally characterized in *C. elegans*.

## Introduction

*Caenorhabditis elegans* is a free-living, microscopic soil nematode. Since its isolation in 1963, this bacterivorous animal acquired the status of model organism in neurobiology (Brenner, 1974). *C. elegans* is easy to cultivate and has a short life cycle of about 3 days at 20°C. Before reaching its adult form, it goes through four larval stages (L1–L4). Hermaphrodites can either self-fertilize or mate with males, a feature that is commonly exploited during high-throughput genetic studies. The publication of its approximately 100 Mb genome in 1998 made *C. elegans* the first multicellular organism to have its entire genome sequenced (The *C. elegans* Sequencing Consortium, 1998). Genome-wide comparison of the predicted *C. elegans* genes and their vertebrate equivalents revealed an unexpected but notable resemblance between their nervous systems (Bargmann, 1998). The *C. elegans* nervous system comprises only 302 small neurons in adult hermaphrodites. It might seem simple at first sight, but appears to be chemically complex, equivalent to most vertebrate nervous systems. Chemical signaling in *C. elegans* occurs through a group of classical neurotransmitters which are important for synaptic communication and includes acetylcholine (ACh), γ-aminobutyric acid (GABA), glutamate, nitric oxide, serotonin, and other monoamines (Brownlee and Fairweather, 1999). These small-molecule neurotransmitters are packed into synaptic vesicles and subsequently released by exocytosis (Gasnier, 2000; Weimer and Jorgensen, 2003; Scalettar, 2006). In addition to classical neurotransmitters, cell-to-cell communication via chemical signaling in *C. elegans* also occurs through neuropeptides. Both bioinformatic predictions and peptidomic analyses demonstrated that the *C. elegans* genome comprises a rich diversity of small neuropeptide bioregulators (Li et al., 1999; Nathoo et al., 2001; Pierce et al., 2001; Husson et al., 2005, 2007a; Li and Kim, 2010). This abundant group of over 250 signaling molecules is derived from neuropeptide precursor genes and outnumbers the classical neurotransmitters. As observed in other animal species, one or multiple mature bioactive neuropeptides are generated out of each preproprotein precursor by proteolytic processing and extensive post-translational modifications (Husson et al., 2005, 2006, 2007b; Husson and Schoofs, 2007). Besides their role in key physiological processes, it seems that *C. elegans* neuropeptides are implicated in the modulation of essentially all behaviors including locomotion, reproduction, social behavior, mechano- and chemosensation, learning and memory (Li and Kim, 2008); and may be important for behavioral adaptation throughout evolution (Avery, 2010). Neuropeptides are primarily thought to act as neuromodulators but can also act as fast neurotransmitters. Despite the lack of a circulatory system in *C. elegans*, a neurohormonal role is also ascribed to neuropeptides, and its nervous system harbors a significant number of peptidergic neurosecretory cells (Hartenstein, 2006). It is assumed that possibly all *C. elegans* neurons synthesize and secrete neuropeptides (Holden-Dye and Walker, 2012). Currently, 119 neuropeptide precursor genes are known which can be subdivided into three major families according to the sequence and structural similarities of their derived peptides. Thirty-one neuropeptide-encoding genes are assigned to the FMRFamide (Phe-Met-Arg-Phe-amide)-like peptide (*flp*) gene family, while 40 genes belong to the family of insulin-like peptide (*ins*) genes. Peptides that bear no resemblance to FMRFamide- or insulin-like peptides are encoded by the family of neuropeptide-like protein (*nlp*) genes. So far, 48 *nlp* precursor genes are known. G protein-coupled receptors (GPCRs) are the principal neuropeptide targets through which intracellular signaling transduction pathways are triggered. GPCRs are defined as seven transmembrane receptors that signal through G proteins. They are found in almost any eukaryotic organism indicating they have an early evolutionary origin (Krishnan et al., 2012). GPCRs have a diverse array of ligands ranging from light, Ca^2+^ and odorants to small molecules such as amino acid residues, nucleotides, peptides, and proteins (Pin, 2000). About 7% of all predicted protein-coding genes in *C. elegans* are GPCRs (Bargmann, 1998; Fredriksson and Schiöth, 2005). Most of them (∼1300) encode nematode-specific chemoreceptors, which are thought to compensate for the absence of visual and auditory systems in *C. elegans* (Thomas and Robertson, 2008). The remaining GPCRs can be classified according to the GRAFS classification system and comprise the Glutamate, Rhodopsin, Adhesion, Frizzled, and Secretin families (Schiöth and Fredriksson, 2005). In this review we will focus on the *C. elegans* neuropeptide GPCRs, which belong to the rhodopsin and secretin families. To date, only a limited number of nematode neuropeptide GPCRs of these families have been deorphanized and functionally characterized.

## Unraveling Neuropeptidergic Signaling in the Model Organism *C. elegans*

The flexible genetic tool box that comes with the use of *C. elegans* as a model has greatly expedited the functional characterization of neuropeptide GPCRs in this organism. Genome-wide RNA interference (RNAi) and mutant analyses have been used to shed light on the behavioral output of neuropeptidergic signaling (Keating et al., 2003; Rual et al., 2004; Ceron et al., 2007). With these techniques, it has become clear that neuropeptides and their receptors influence many if not all of the worm’s behaviors. This conclusion is further supported by results from genetic studies on orphan *C. elegans* GPCRs. Recently, Jee et al. (2012) generated mutants for the SEB-3 receptor, an orphan corticotropin-releasing factor (CRF)-related GPCR, and showed that it is strongly implicated in the worm’s stress response and ethanol tolerance. This is a perfect example of an earlier finding that several neuropeptide pathways are involved in *C. elegans* responses to ethanol (Mitchell et al., 2010). A genome-wide RNAi study of predicted *C. elegans* GPCRs was performed by Keating et al. (2003) amongst others, which were able to identify a number of neuropeptide receptors involved in reproduction and locomotion. *In vivo* localization of neuropeptide signaling components is facilitated by the worm’s transparency and its simple but well-defined anatomy. The adult hermaphrodite has exactly 959 somatic nuclei ordered in fully differentiated tissues (Sulston et al., 1983; WormAtlas et al., 2002–2012). The developmental origin of every *C. elegans* neuron and the wiring diagram of its roughly 7000 synapses have been completely mapped (White et al., 1986; Jarrell et al., 2012). Using selective promoters that target gene expression to a cell or tissue of interest, combined with laser ablation and cell imaging techniques, gene function and neural activity can be studied at the level of individual neurons, of which several examples are described below. Together, these powerful molecular and genetics tools enable the dissection of neural networks underlying the neuropeptidergic regulation of behavior with single cell resolution.

## Neuropeptide GPCRs in *C. elegans*

Since the publication of the *C. elegans* genome, many predictions have been made about the number of neuropeptide GPCRs it contains. These predictions are usually based on sequence similarities to vertebrate and insect neuropeptide GPCRs (Bargmann, 1998; Fredriksson and Schiöth, 2005). Compared to other *C. elegans* GPCRs, neuropeptide GPCRs appear to be less closely related to their vertebrate counterparts. This agrees with the apparently low conservation of the *C. elegans* neuropeptides (Bargmann, 1998). A crucial step in the characterization of a predicted neuropeptide GPCR is the identification of its natural ligand(s). For this purpose, a reverse pharmacology approach (see Finding Neuropeptide Ligands for Orphan GPCRs) can be applied (Mertens et al., 2004; Beets et al., 2011). Another way to predict neuropeptide GPCRs is to use all deorphanized neuropeptide GPCRs as a seeding set in a Multiple Expectation Maximization for Motif Elicitation/Motif Alignment and Search Tool (MEME/MAST) analysis. Doing so, a list of 125 potential neuropeptide receptors could be obtained (Janssen et al., 2010). Since this prediction, the number of deorphanized neuropeptide GPCRs has increased from 6 to 23. We enhanced this MEME/MAST analysis by use of an updated seeding set containing all newly deorphanized neuropeptide GPCRs and merged our predictions with the list of neuropeptide GPCRs from WormAtlas et al. (2002–2012). Manual verification of every receptor ensured a reliable, revised list of potential neuropeptide GPCRs in *C. elegans* (Table 1).

**Table 1:** **List of orphan and deorphanized neuropeptide GPCRs in *C. elegans* (adapted from WormAtlas et al., 2002–2012)**.

Receptor group	Gene sequence name	Protein	WormBase ID	Ligand-encoding precursor gene (Ligand^a^)	Putative role in	Reference
**RHODOPSIN FAMILY OF GPCRs**						
Neuropeptide Y/RFamide-like receptors	C39E6.6	NPR-1	WP:CE06941	*flp-18* (FLP-18-1, FLP-18-2, FLP-18-3, FLP-18-4, FLP-18-5, FLP-18-6); *flp-21* (FLP-21)	Feeding behavior; aerotaxis; thermal avoidance; ethanol tolerance; innate immunity	de Bono and Bargmann (1998), Kubiak et al. (2003), Rogers et al. (2003), Davies et al. (2004), Cheung et al. (2005), Rogers et al. (2006), Gloria-Soria and Azevedo (2008), Styer et al. (2008), Glauser et al. (2011), Milward et al. (2011)^c^
	T05A1.1a	NPR-2a	WP:CE32924	/	Fat storage; locomotion^b^	Keating et al. (2003), Cohen et al. (2009)^c^
	T05A1.1b	NPR-2b	WP:CE32925	/	
	C10C6.2	NPR-3	WP:CE08056	*flp-15* (FLP-15-1, FLP-15-2)	Locomotion^b^	Keating et al. (2003), Kubiak et al. (2003)^c^
	C16D6.2	NPR-4	WP:CE37317	*flp-1* (FLP-1-6); *flp-4* (FLP-4-2); *flp-18* (FLP-18-1, FLP-18-2, FLP-18-3, FLP-18-4, FLP-18-5, FLP-18-6)	Fat storage; olfaction; foraging; reproduction^b^	Keating et al. (2003), Lowery et al. (2003), Cohen et al. (2009)^c^
	Y58G8A.4a	NPR-5a	WP:CE33345	*flp-1* (FLP-1-2); *flp-3* (FLP-3-1, FLP-3-3, FLP-3-6, FLP-3-8); *flp-18* (FLP-18-1, FLP-18-2, FLP-18-3, FLP-18-4, FLP-18-5, FLP-18-6); *flp-21* (FLP-21)	Fat storage; dauer formation	Kubiak et al. (2008), Cohen et al. (2009)^c^
	Y58G8A.4b	NPR-5b	WP:CE36962	*flp-1* (FLP-1-2); *flp-3* (FLP-3-1, FLP-3-3, FLP-3-6, FLP-3-8); *flp-4* (FLP-4-2); *flp-18* (FLP-18-1, FLP-18-2, FLP-18-3, FLP-18-4, FLP-18-5, FLP-18-6); *flp-21* (FLP-21)		Lowery et al. (2003), Kubiak et al. (2008)^c^
	F41E7.3	NPR-6	WP:CE31509 WP:CE31509	*flp-18* (FLP-18-3, FLP-18-6); *flp-21* (FLP-21)	Reproduction^b^	Keating et al. (2003); Lowery et al. (2003); ^c^
	F35G8.1	NPR-7	WP:CE39498	/	Fat storage; locomotion^b^	Keating et al. (2003), Cohen et al. (2009)^c^
	C56G3.1a	NPR-8a	WP:CE04283	/	/	^c^
	C56G3.1b	NPR-8b	WP:CE30923	/	/	^c^
	C53C7.1a C53C7.1b	NPR-10a NPR-10b	WP:CE19767 WP:CE36989	*flp-3* (FLP-3-1, FLP-3-3, FLP-3-5, FLP-3-6, FLP-3-7, FLP-3-8); *flp-18* (FLP-18-1, FLP-18-3, FLP-18-4, FLP-18-5, FLP-18-6)	/ /	Lowery et al. (2003)^c^
	C25G6.5	NPR-11	WP:CE47199	*flp-*1 (FLP-1-6); *flp-5* (FLP-5-2); *flp-14* (FLP-14); *flp-18* (FLP-18-3); *flp-21* (FLP-21); *nlp-1* (MDANAFRMSFamide)	Local search behavior; olfactory adaptation, reproduction^b^	Keating et al. (2003), Lowery et al. (2003), Chalasani et al. (2010)^c^
	T22D1.12	NPR-12	WP:CE17256	/	Reproduction^b^	Rual et al. (2004)^c^
	ZC412.1	NPR-13	WP:CE35920	/	/	^c^
	W05B5.2	NPR-14	WP:CE42751	/	/	^c^
	T07D4.1	NPR-20	WP:CE46449	/	/	^c^
	T23C6.5	NPR-21	WP:CE35783	/	/	^c^
	C02B8.5	FRPR-1	WP:CE47103	/	/	
	C05E7.4	FRPR-2	WP:CE46227	/	/	
	C26F1.6	FRPR-3	WP:CE06880	*flp-7* (FLP-7-1, FLP-7-2, FLP-7-3); *flp-11* (FLP-11-1)	Reproduction^b^	Keating et al. (2003), Mertens et al. (2004)^c^
	C54A12.2	FRPR-4	WP:CE36809	/	/	^c^
	C56A3.3a	FRPR-5a	WP:CE45431	/	/	^c^
	C56A3.3b	FRPR-5b	WP:CE45452	/	/	^c^
	F21C10.12	FRPR-6	WP:CE32868	/	/	^c^
	F53A9.5	FRPR-8	WP:CE43844	/	/	^c^
	F53B7.2a	FRPR-9a	WP:CE44099	/	/	^c^
	F53B7.2b	FRPR-9b	WP:CE44063	/	/	^c^
	K06C4.8	FRPR-11	WP:CE11816	/	/	^c^
	K06C4.9	FRPR-12	WP:CE29506	/	/	^c^
	K07E8.5	FRPR-14	WP:CE35992	/	/	^c^
	K10C8.2	FRPR-15	WP:CE42140	/	/	^c^
	R12C12.3	FRPR-16	WP:CE02848	/	/	^c^
	T14C1.1	FRPR-17	WP:CE34212	/	/	
	T19F4.1a	FRPR-18a	WP:CE29348	*flp-2* (FLP-2-1, FLP-2-2)	/	Mertens et al. (2005)^c^
	T19F4.1b	FRPR-18b	WP:CE29349		/	
	Y41D4A.8	FRPR-19	WP:CE21846	/	/	^c^
	C30B5.5	FRPR-20	WP:CE02524	/	/	^c^
	C09F12.3	C09F12.3	WP:CE33973	/	/	^d^
	D1014.2	D1014.2	WP:CE44520	/	/	
Somatostatin and galanin-like receptors	ZK455.3	NPR-9	WP:CE03814	/	Fat storage; roaming behavior	Bendena et al. (2008)^c^
	F56B6.5a	NPR-16a	WP:CE31186	/	Lipid metabolism^b^	Ashrafi et al. (2003)^c^
	F56B6.5b	NPR-16b	WP:CE39375	/	
	C06G4.5	NPR-17	WP:CE38997	/	/	^c^
	C43C3.2a	NPR-18a	WP:CE01524	/	/	^c^
	C43C3.2b	NPR-18b	WP:CE47537	/	/	^c^
	C43C3.2c	NPR-18c	WP:CE47497	/	/	^c^
	C43C3.2d	NPR-18d	WP:CE47523	/	/	^c^
	C43C3.2e	NPR-18e	WP:CE47594	/	/	^c^
	C43C3.2f	NPR-18f	WP:CE47636	/	/	^c^
	C43C3.2g	NPR-18g	WP:CE47570	/	/	^c^
	R106.2	NPR-24	WP:CE39612	/	/	^c^
	T02E9.1	NPR-25	WP:CE13062	/	Locomotion^b^	Keating et al. (2003)^c^
	T02D1.6	NPR-26	WP:CE30684	/	/	^c^
	F42C5.2	NPR-27	WP:CE47648	/	/	^c^
	F55E10.7	NPR-28	WP:CE40072	/	/	
	ZC84.4	NPR-29	WP:CE24711	/	/	
	H10E21.2	NPR-30	WP:CE43737	/	/	
	Y116A8B.5	NPR-32	WP:CE46735	/	/	
	Y54E2A.1	Y54E2A.1	WP:CE31259	/	/	^c^
	C17H11.1	C17H11.1	WP:CE29584	/	/	^d^
	C24B5.1	C24B5.1	WP:CE36981	/	/	^d^
	F57A8.4	F57A8.4	WP:CE05985	/	/	^c^
	W10C4.1	W10C4.1	WP:CE39426	/	/	^d^
Tachykinin (neurokinin)-like receptors	C38C10.1	TKR-1	WP:CE44282	/	Lipid metabolism^b^	Ashrafi et al. (2003)^c^
	AC7.1a	TKR-3a	WP:CE38261	/	Reproduction^b^	Kamath et al. (2003), Simmer et al. (2003), Green et al. (2011)^c^
	AC7.1b	TKR-3b	WP:CE38262	/	
	T27D1.3	NPR-15	WP:CE43181	/	/	^c^
	Y59H11AL.1a	NPR-22a	WP:CE31260	*flp-1* (FLP-1-6); *flp-7* (FLP-7-1, FLP-7-2, FLP-7-3, FLP-7-4); *flp-9* (FLP-9); *flp-11* (FLP-11-1, FLP-11-2, FLP-11-3); *flp-13* (FLP-13-4); *flp-22* (FLP-22)	/	Mertens et al. (2006)^c^
	Y59H11AL.1b	NPR-22b	WP:CE38456	/	/	^c^
	C49A9.7	C49A9.7	WP:CE16937	/	/	^c^
	C50F7.1a	C50F7.1a	WP:CE33574	/	/	^c^
	C50F7.1b	C50F7.1b	WP:CE04239	/	/	^c^
	F31B9.1	NPR-33	WP:CE17727	/	/	^c^
	T11F9.1a	T11F9.1a	WP:CE47396	/	/	^c^
	T11F9.1b	T11F9.1b	WP:CE47310	/	/	^c^
Cholecystokinin/gastrin-like receptors	T23B3.4	CKR-1	WP:CE45656	/	Reproduction^b^	Rual et al. (2004)^c^
	Y39A3B.5c/b	CKR-2a	(GenBank database accession number EU346943)	*flp-1* (FLP-1-1); *nlp-12* (DYRPLQFamide, DGYRPLQFamide); *nlp-13* (SSSMYDRDIMSFamide); *nlp-14* (ALDGLDGSGFGFD)	Locomotion; fat storage; amylase activity/secretion	Janssen et al. (2008a), Hu et al. (2011)^c^
	Y39A3B.5c/d	CKR-2b	(GenBank database EU346944 accession number)			
Gonadotropin	F54D7.3a	GNRR-1a	WP:CE17102	*nlp-47* (NLP-47)	Reproduction^b^	Lindemans et al. (2009a)^c^
releasing hormone, oxytocin, vasopressin-like receptors		F45D7.3b	GNRR-1b	WP:CE46861	/	/	^c^
	C15H11.2a	GNRR-2a	WP:CE08179	/	/	
	C15H11.2b	GNRR-2b	WP:CE45186	/	/	
	ZC374.1	GNRR-3	WP:CE40886	/	/	
	C41G11.4a	GNRR-4a	WP:CE29716	/	/	
	C41G11.4b	GNRR-4b	WP:CE30896	/	/	
	C41G11.4c	GNRR-4c	WP:CE35428	/	/	
	H22D07.1	GNRR-5	WP:CE33129	/	/	
	F13D2.2	GNRR-6	WP:CE03194	/	Locomotion^b^	Kamath et al. (2003)
	F13D2.3	GNRR-7	WP:CE33996	/	/	
	Y105C5A.23	GNRR-8	WP:CE24056	/	Reproduction^b^	Ceron et al. (2007)
	T07D10.2	NTR-1	WP:CE13377	*ntc-1* (CFLNSCPYRRYamide)	Gustatory associative learning, reproduction	Beets et al. (2012), Garrison et al. (2012)^c^
	F14F4.1	NTR-2	WP:CE17670	/	/	^c^
Neurotensin, neuromedin U, growth hormone secretagogue, thyrotropin releasing hormone-like receptors	C48C5.1	NMUR-1	WP:CE45664	/	/	^c^
	K10B4.4	NMUR-2	WP:CE38395	*nlp-44* (AFFYTPRIamide)	/	Lindemans et al. (2009b)^c^
	F02E8.2a	NMUR-3a	WP:CE33990	/	/	^c^
	F02E8.2b	NMUR-3b	WP:CE07017	/	/	^c^
	C30F12.6	NMUR-4	WP:CE16886	/	Reproduction^b^	Ceron et al. (2007)
	F57H12.4	FRPR-10	WP:CE43724	/	/	^c^
	F57B7.1a	DMSR-1a	WP:CE05989	/	/	^e^
	F57B7.1b	DMSR-1b	WP:CE31009	/	/	^e^
	C46F4.1a C46F4.1b	EGL-6a EGL-6b	WP:CE04219 WP:CE43400	*flp-10* (FLP-10); *flp-17* (FLP-17-1, FLP-17-2)	Reproduction	Trent et al. (1983), Ringstad and Horvitz (2008)
	B0563.6a	B0563.6a	WP:CE29551	/	/	^e^
	B0563.6b	B0563.6b	WP:CE33513	/	/	^e^
	B0563.6c	B0563.6c	WP:CE41751	/	/	^e^
	R03A10.6	SPRR-1	WP:CE43810	/	/	^d^
	F42D1.3	SPRR-2	WP:CE31511	/	/	^d^
	F39B3.2	FRPR-7	WP:CE30978	/	/	^d^
	K03H6.5	K03H6.5	WP:CE39585	/	/	^d^
/	C48C5.3	AEXR-3	WP:CE04226	/	/	^c^
/	T10E10.3	T10E10.3	WP:CE35766	/	/	^d^
/	T21H3.5	T21H3.5	WP:CE13906	/	/	^d^
/	Y70D2A.1	Y70D2A.1	WP:CE34231	/	/	^d^
/	ZK1307.7a	ZK1307.7a	WP:CE37863	/	/	^d^
/	ZK1307.7b	ZK1307.7b	WP:CE46539	/	/	^d^
/	B0034.5	B0034.5	WP:CE45030	/	/	^d^
/	B0334.6	B0334.6	WP:CE30473	/	/	^d^
**SECRETIN FAMILY OF GPCRs**						
/	C18B12.2	SEB-3	WP:CE23557CE23557		Locomotion; stress response; ethanol tolerance	Jee et al. (2012)
	B0457.1a	LAT-1a	WP:CE02945	/	/	^f^
	B0457.1b	LAT-1b	WP:CE32789	/	/	^f^
	B0286.2a	LAT-2a	WP:CE36968	/	/	^f^
	C18B12.2	C18B12.2	WP:CE23557	/	/	^f,g^
	ZK643.3a	SECR-1a	WP:CE33750	/	/	^f,g^
	ZK643.3b	SECR-1b	WP:CE01112	/	/	^f,g^
	C13B9.4a	PDFR-1a	WP:CE30860	*pdf-1* (PDF-1a, PDF-1b); *pdf-2* (PDF-2)	Locomotion; reproduction	Janssen et al. (2008b), Meelkop et al. (2012)
	C13B9.4b	PDFR-1b	WP:CE370787			
	C13B9.4c	PDFR-1c	WP:CE37088			

*GPCRs are ranked according to the GRAFS classification system. Deorphanized neuropeptide GPCRs are marked in gray. Underlined neuropeptides are most potent to be the endogenous ligands (^a^see Table A1 in Appendix for neuropeptide ligand sequences). ^b^Putative roles supported by RNAi. Neuropeptide GPCRs are predicted in ^c^Janssen et al. (2010), ^e^Hewes and Taghert (2001), ^f^Harmar (2001), and ^g^Cardoso et al. (2006). ^d^To complete the list of neuropeptide GPCR we performed a MEME/MAST analysis as described in Janssen et al. (2010)*.

All predicted neuropeptide GPCRs can be grouped in the rhodopsin and secretin families according to the GRAFS classification system. Rhodopsin GPCRs are subdivided based on their resemblance to insect and mammalian neuropeptide GPCRs. The neuropeptide Y (NPY)/RFamide-like receptor family, containing 41 receptors, represents the best characterized group. Twelve of its representatives have been deorphanized and all are activated by FMRFamide like peptides (NPR-1, NPR-3, NPR-4, NPR-5a/b, NPR-6, NPR-10a/b, NPR-11, FRPR-3, and FRPR-18a/b). Their corresponding signaling pathways are involved in a multitude of functions such as locomotion, feeding, energy metabolism, and reproduction. So far, none of the 24 receptors belonging to the somatostatin and galanin-like receptor group have been deorphanized. Only RNAi phenotypes with respect to locomotion and fat metabolism have been observed for this poorly studied group. The tachykinin (neurokinin)-like receptor group contains 12 receptors. Mertens et al. (2006) deorphanized one of these receptors, namely NPR-22a. Remarkably, this GPCR was not activated by the predicted *C. elegans* tachykinin-like peptide but by a handful of FMRFamide-related peptides (FaRPs). The cholecystokinin (CCK)/gastrin-like receptor and gonadotropin releasing hormone (GnRH), oxytocin (OT), vasopressin (VP)-like receptor groups contain 3 and 14 receptors, respectively. Deorphanization of CKR-2a/b and GNRR-1a supports the theory of receptor-ligand coevolution (Janssen et al., 2010). Although no clear CCK or sulfakinin orthologs could be identified through *in silico* searches, library-based screening led to the identification of NLP-12a and NLP-12b as the endogenous ligands of CKR-2a/b. Alignment of these peptides to vertebrate CCK/gastrin hormones and arthropod sulfakinins revealed their similarity. The endogenous ligand of GNRR-1a was found using an *in silico* approach. GNRR-1a and its ligand, NLP-47, are both involved in reproduction as shown by RNAi experiments (Lindemans et al., 2009a). The VP/OT-like receptor NTR-1 has only recently been identified and deorphanized. The VP/OT-related signaling system is involved in gustatory associative learning and male reproduction (Beets et al., 2012; Garrison et al., 2012). The group of neurotensin, neuromedin U (NMU), growth hormone secretagogue, and thyrotropin releasing hormone (TRH)-like receptors contains 17 receptors, of which three have been deorphanized: NMUR-2 and EGL-6a/b. Only the EGL-6a/b receptors are functionally characterized and they proved to be involved in the regulation of egg-laying (Ringstad and Horvitz, 2008). The secretin family of GPCRs contains nine receptors. Of these, the three pigment dispersing factor (PDF) GPCRs are deorphanized and play a role in locomotion and egg-laying (Janssen et al., 2008a; Meelkop et al., 2012).

## G Protein Signaling in *C. elegans*

**Figure 1:**
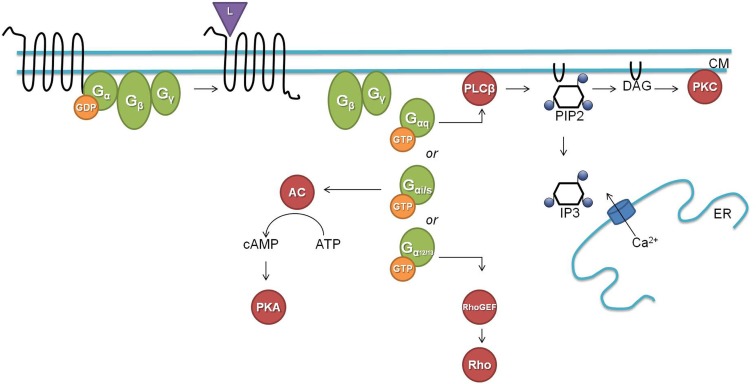
**The classical G protein signaling pathway (L, ligand; CM, cell membrane; GDP, guanosine diphosphate; GTP, guanosine triphosphate; AC, adenylate cyclase; cAMP, cyclic adenosine monophosphate; ATP, adenosine triphosphate; PKA, protein kinase A; PLCβ, phospholipase Cβ; PIP2, phosphatidylinositol 4,5-bisphosphate; DAG, diacylglycerol; IP3, inositol-1,4,5-trisphosphate; ER, endoplasmatic reticulum; PKC, protein kinase C; GEF, guanine nucleotide exchange factor)**.

G protein-coupled signaling pathways are highly conserved among *C. elegans* and mammals. In the classical G protein signaling pathway (Figure 1), the inactive receptor is bound to the heterotrimeric Gαβγ protein. Upon binding of an activating ligand, the receptor changes its confirmation and acts as a guanine nucleotide exchange factor (GEF) by catalyzing the release of GDP and binding of GTP by the Gα subunit. The now activated heterotrimeric Gαβγ protein dissociates from the receptor and splits into a Gα-GTP and a Gβγ subunit. Gα-GTP regulates different effectors depending on the Gα subtype (Gα_s_, Gα_i/o_, Gα_q_, and Gα_12/13_). Gα_q_ is known for its activation of phospholipase Cβ (PLCβ), which splits phosphatidylinositol 4,5-bisphosphate (PIP2) into diacylglycerol (DAG) and inositol-1,4,5-trisphosphate (IP3). Binding of IP3 to IP3 dependent calcium channels leads to an increase in calcium, and DAG will bind and activate protein kinase C (PKC). Gα_s_ and Gα_i/o_ act through adenylyl cyclase by stimulating (Gα_s_) or inhibiting (Gα_i/o_) its activity and thereby regulating the concentration of cyclic AMP, which activates protein kinase A (PKA). Gα_12/13_ activates Rho dependent pathways. The Gβγ subunit also regulates certain downstream effectors such as ion channels and PLCβ. G protein signaling is terminated by internalization of the GPCR, which is initiated by phosphorylation through GPCR kinases (GRKs; Ritter and Hall, 2009).

*C. elegans* has homologs for most of the above described G proteins and downstream second messengers. The worm has 21 Gα, 2 Gβ (GPB-1 and GPB-2), and 2 Gγ (GPC-1 and GPC-2) proteins. GPB-1 and GPC-2 seem to be mediators in the classical G protein signaling as the homologs of Gβ and Gγ respectively. For each of the four mammalian Gα subtypes there is a homologous Gα protein in *C. elegans* [GSA-1 (Gα_s_), GOA-1 (Gα_i/o_), EGL-30 (Gα_q_), and GPA-12 (Gα_12/13_)]. The remaining *C. elegans* Gα subtypes are believed to be specific for chemosensory GPCRs (Jansen et al., 1999; Bastiani and Mendel, 2006). EGL-30 and GSA-1 are the only Gα proteins for which the conservation of their downstream targets has been demonstrated. The classical role of the EGL-30 Gα_q_ protein is intensively studied in neuromuscular junctions where it stimulates the release of the neurotransmitter ACh. EGL-30 binds and activates EGL-8, the PLCβ homolog, which splits PIP2 into IP3 and DAG. In neuromuscular junctions, DAG binds to UNC-13 which regulates synaptic vesicle release of ACh through syntaxin (Lackner et al., 1999). IP3 on the other hand can bind to the IP3 dependent calcium channel ITR-1 which leads to a calcium response (Bastiani et al., 2003; Baylis and Vázquez-Manrique, 2012). The Gα_s_ protein homolog GSA-1 seems to function through the adenylate cyclase ACY-1. GSA-1 is an essential protein but constitutive activation of GSA-1 in the presence of ACY-1 causes neurodegeneration (Korswagen et al., 1997; Berger et al., 1998). Constitutive expression of rat Gα_s_ correspondingly causes the same neurodegenerative phenotype.

## Finding Neuropeptide Ligands for Orphan GPCRs

To find the activating ligand(s) of a GPCR, a reverse pharmacology approach can be applied. In this approach, the orphan GPCR is expressed in a heterologous expression system. Often Chinese hamster ovary (CHO) or human embryonic kidney (HEK) cells are the recombinant systems of choice because of their ease of use and proven history of functional GPCR expression (Szekeres, 2002). Subsequently, receptor expressing cells are challenged with a library of compounds and activation of the GPCR of interest is measured. The compound library is usually compiled based on bioinformatic predictions and peptidomic analyses of RP-HPLC fractions of a tissue extract (Beets et al., 2011). In the past few years, several successful strategies have been developed for receptor deorphanization (Mertens et al., 2004; Beets et al., 2011). One of the most frequently used methods is probably the calcium mobilization assay based on the detection of intracellular calcium that is released from storage sites upon receptor activation. This method is often combined with the co-expression of a promiscuous G protein, such as the Gα_16_ subunit, which can direct the intracellular signaling cascade of the activated receptor through a calcium flux (Offermanns and Simon, 1995). Alternatively, chimeric G proteins can be used to lead the signal cascade to a pathway of choice (Milligan and Rees, 1999). The resulting calcium flux can then be detected by bioluminescent proteins such as aequorin, or by fluorescent calcium indicators (e.g., Fluo-4). In the bioluminescent assay, cells expressing the apoaequorin protein are charged prior to the assay with the cofactor coelenterazine to form a calcium-sensitive aquorin complex. When calcium binds to aequorin, the complex is oxidized and blue light is omitted. Similar to the luminescence assay, receptor expressing cells can be loaded with a fluorophore, of which the fluorescence increases upon binding of calcium (Mertens et al., 2004). Thanks to the development of automated systems for simultaneous compound addition and signal detection in various well-plate formats, such as the FLEXstation^®^ (Molecular Devices, CA, USA) fluorescent plate reader, calcium mobilization methods can be used in high-throughput screening assays. Once the activating ligand(s) of a receptor are found, the endogenous Gα signaling protein is identified by omitting the promiscuous Gα_16_ protein. Coupling of a receptor with Gα_q_, Gα_s_, or Gα_i_ can be visualized by respectively measuring the calcium increase or cAMP in-/decrease.

## Characterized Neuropeptidergic Signaling Pathways

### NPR-1 signaling: Inhibition of aggregation and aerotaxis

The neuropeptide receptor 1 (NPR-1) was the first neuropeptide GPCR to be deorphanized in *C. elegans* (Kubiak et al., 2003; Rogers et al., 2003). This receptor shows homology to the vertebrate NPY receptor family that is implicated in a variety of physiological processes such as food intake and stress (Heilig, 2004; Arora and Anubhuti, 2006). In the nematode *C. elegans*, NPR-1 is involved in a multitude of functions such as food-dependent behaviors, thermal avoidance, ethanol tolerance, and innate immunity (de Bono and Bargmann, 1998; Davies et al., 2004; Gray et al., 2004; Cheung et al., 2005; Rogers et al., 2006; Gloria-Soria and Azevedo, 2008; Styer et al., 2008; Glauser et al., 2011; Milward et al., 2011; Jang et al., 2012).

**Figure 2:**
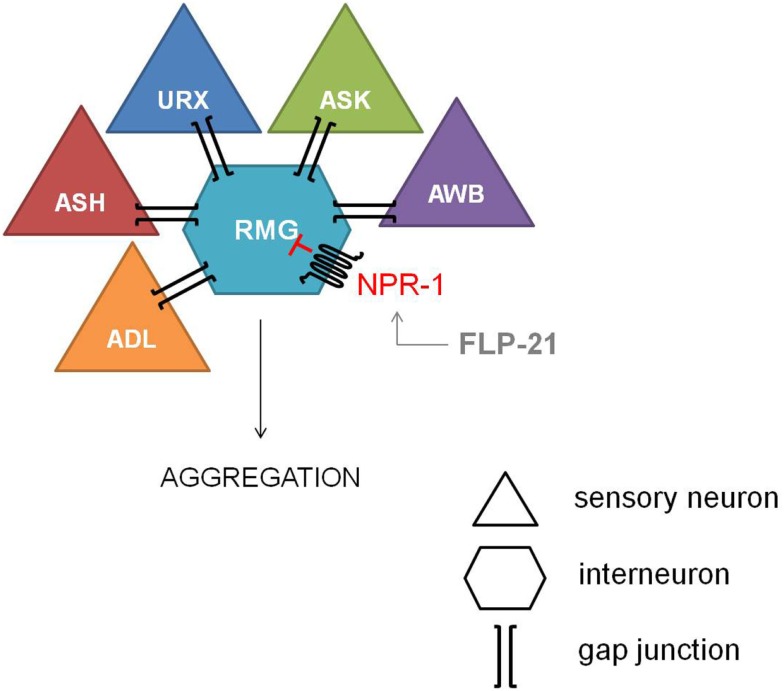
**Inhibiton of the RMG inter/motor neuron by NPR-1**. The RMG neuron is the hub of a gap junction network connecting the ADL, ASH, URX, ASK, and AWB sensory neurons, which are known to trigger aggregation. NPR-1 inhibits this gap junction driven activation of RMG (figure adapted from Macosko et al., 2009).

The most explicit function of NPR-1 was elucidated with the observation of aggregating and solitary feeders in wild type isolates of *C. elegans* (de Bono and Bargmann, 1998). This behavioral difference could be attributed to a single amino acid difference. Aggregating isolates carry an *npr-1* Phe-215 allele whereas solitary feeders possess an *npr-1* Val-215 allele. Since a functional null mutation of *npr-1* converts the solitary wild type N2 lab strain into an aggregating one, NPR-1 activity is suggested to suppress aggregating behavior. The RMG inter/motor neuron seems to be the cellular hub of this NPR-1 mediated feeding behavior, as demonstrated by the full rescue of the solitary behavior through RMG-specific expression of NPR-1 in an *npr-1* knockout mutant (Macosko et al., 2009). The RMG neuron is the hub of a gap junction network that connects five sensory neurons which are known to trigger aggregation, while NPR-1 inhibits this gap junction driven activation of RMG (Figure 2).

Reduced NPR-1 activity in the RMG-hub-and-spoke circuit also contributes to thermal avoidance and sex-specific pheromone responses in *C. elegans*. Deletion of the NPR-1 receptor increases the threshold for heat avoidance, and cell-specific rescue of *npr-1* demonstrates the role of the RMG interneuron in the regulation of heat avoidance behavior (Glauser et al., 2011). Similarly, RMG-specific rescue of *npr-1* restores pheromone avoidance defects in the *npr-1* mutant background (Jang et al., 2012). Therefore, the RMG neural network can be considered a multifunctional sensory circuit that uses neuropeptide GPCR signaling amongst others to coordinate behavioral output.

In insects and mollusks FaRPs are reported as ligands for NPR-1-like receptors (Tensen et al., 1998; Feng et al., 2003). In 2003, two independent groups were able to deorphanize the NPR-1 receptor by using *C. elegans* and other invertebrate FaRPs. Both FLP-21 and FLP-18 peptides activated the solitary Val-215 receptor. The social Phe-215 receptor variant could only be activated by FLP-21. NPR-1 signaling occurs through a Gα_i/o_ type of G protein (Kubiak et al., 2003; Rogers et al., 2003). Deorphanization of the NPR-1 receptor supports its role in repressing aggregation, since the solitary Val-215 receptor variant displayed higher binding and functional activity than the Phe-215 receptor variant.

**Figure 3:**
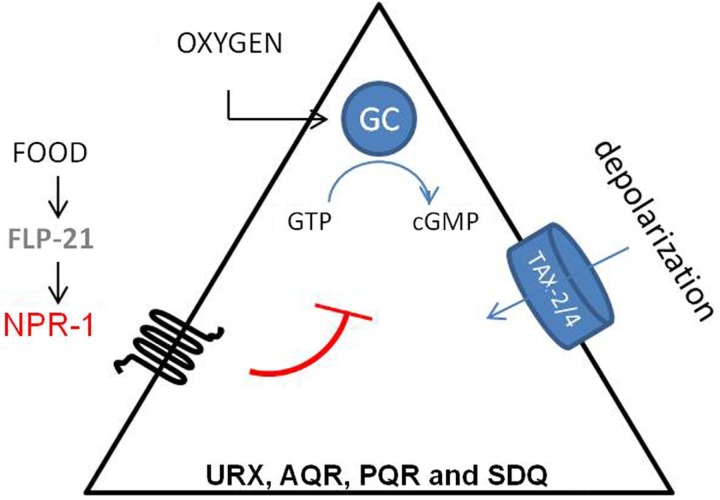
**Regulation of aerotaxis by NPR-1**. *C. elegans* detects oxygen through the URX, PQR, AQR, SDQ neurons. Oxygen sensing in these neurons is mediated by soluble guanylate cyclase homologs (sGC-35 and sGC-36). When ambient oxygen levels decrease, cGMP levels rise and the cGMP gated TAX-2/TAX-4 ion channel opens, which leads to the depolarization of the neurons. Activation of NPR-1 in the presence of food inhibits this activation (Cheung et al., 2005; Chang et al., 2006).

Besides its role in feeding behavior, NPR-1 also regulates aerotaxis (Figure 3). *C. elegans* exhibits a strong behavioral preference for 5–12% oxygen, avoiding higher and lower oxygen levels. Oxygen levels are sensed by the URX, PQR, AQR, and SDQ neurons (Gray et al., 2004). Oxygen sensing in these neurons is mediated by soluble guanylate cyclase homologs (GCY-35 and GCY-36). When ambient oxygen levels decrease, cGMP levels rise and the cGMP gated TAX-2/TAX-4 ion channel opens, leading to the depolarization of the neurons. Activation of NPR-1 in the presence of food inhibits the activation of these neurons (Cheung et al., 2005; Chang et al., 2006; Rogers et al., 2006). Oxygen binding globins such as GLB-5 further tune the behavioral responses to varying oxygen concentrations, and this effect is again modified by the NPR-1 receptor (Persson et al., 2009). In addition, it has also been shown that the NPR-1 expressing neurons AQR, PQR, and URX contribute to the enhancement of the worm’s sensory perception under hypoxic conditions (Pocock and Hobert, 2010). PQR, AQR, and URX were recently reported to act as tonic receptors that cause long-lasting changes in neural circuit activity that sets *C. elegans* behavior according to ambient oxygen concentrations (Busch et al., 2012), which is also reflected in optimal foraging strategies (Milward et al., 2011). A Ca^2+^ relay involving the L-type voltage-gated Ca^2+^ channel subunit EGL-19, the ryanodine receptor UNC-68, and the inositol-1-4,5-trisphosphate receptor ITR-1 mediate tonic signaling from AQR, PQR, and URX, evoking continuous neuropeptide release.

The role of NPR-1 in the worm’s innate immunity was elucidated by Styer et al. (2008), who uncovered an immune inhibitory function for this receptor. Mutations in the *npr-1* gene directly affect the expression of innate immunity markers, suggesting that neuropeptide GPCRs participate in the neuronal regulation of immune responses. In addition, polymorphisms in the *npr-1* gene have been correlated with the worm’s pathogen avoidance and susceptibility (Aballay, 2009; Reddy et al., 2009, 2011).

### RFamide-like receptor signaling: FLP-18 signaling through NPR-4 and NPR-5

Both a reverse pharmacology study expressing orphan receptors in CHO cells and an independent *Xenopus laevis* oocyte assay demonstrated that the *flp-18*-encoded peptides are the most potent ligands of NPR-5a and NPR-5b, the splice variants of *npr-5* (Kubiak et al., 2008; Cohen et al., 2009). The latter study also showed that another member of the GPCR rhodopsin family, NPR-4, is also activated by FLP-18 peptides (Cohen et al., 2009), which in addition to their activation of NPR-1 (Kubiak et al., 2003) indicates these are widely deployed ligands of GPCRs. NPR-5a and NPR-5b seem to transduce the FLP-18 signal mainly through a Gα_q_ type G protein, while NPR-4 might use a different cellular signaling machinery.

**Figure 4:**
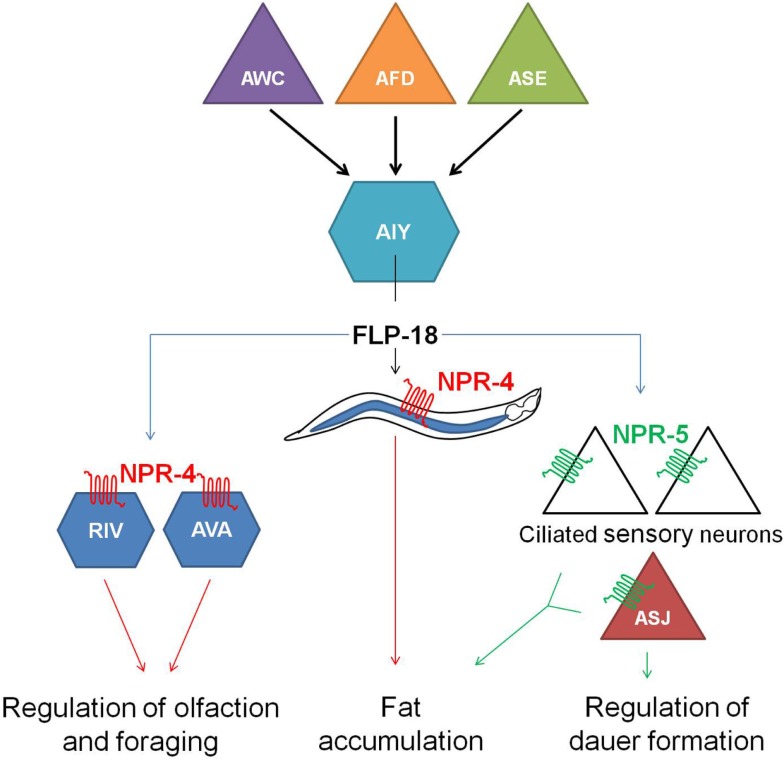
**Hypothetical model in which the detection of nutrition by sensory neurons (AWC, AFD, and ASE) is coupled to the release of FLP-18 neuropeptides from AIY interneurons and subsequent signaling through the RFamide-like receptors NPR-4 and NPR-5**. By acting on NPR-4 in the intestine and NPR-5 in ciliated neurons, FLP-18 neuropeptides control fat storage. Signaling through NPR-4 in RIV and AVA neurons also modulates responses to odor and foraging behavior. Another food-dependent decision, dauer formation, is regulated by FLP-18 action on NPR-5 in the ASJ neurons (figure adapted from Cohen et al., 2009).

*flp-18(db99)* loss-of-function mutants display chemosensory, dauer formation, and foraging defects, accumulate excess intestinal fat and exhibit reduced aerobic metabolism. Distinct subsets of these phenotypes are phenocopied by *npr-4(tm1782)* and *npr-5(ok1583)* deletion mutants. Each one of the FLP-18 receptors regulates fat metabolism in response to the release of FLP-18 peptides from AIY and RIG interneurons in the head, some of the multiple expression sites of *flp-18*. NPR-4 mediated regulation of intestinal fat occurs at the level of the gut, while NPR-5 modulates the activity of a number of amphid sensory neurons. FLP-18 neurohormones released from AIY interneurons act on NPR-4 in AVA and RIV interneurons and appear to be implicated in odor responses and foraging behavior. The chemosensory ASJ neurons regulate dauer formation through activation of NPR-5. All of these observations led to the proposition of a model (Figure 4) in which sensory detection of nutritional availability is coupled to adequate responses such as foraging behavior and metabolic alterations via RFamide-like receptor signaling (Cohen et al., 2009).

### Off-food search behavior: Feedback signaling through NPR-11

Characterization of the neuropeptide GPCR NPR-11 is a good example of how the knowledge of the entire neuronal wiring diagram makes *C. elegans* a favorable model organism. When worms are removed from a food source, they display a local search behavior characterized by increased turning rates during the first 15 min. This behavior is known to depend on the activity of the AWC olfactory neurons, which release both glutamate and the neuropeptide NLP-1. Glutamate is necessary for increased turning rates during the off-food search behavior of the worm, a behavioral change that is also observed in knockout mutants of *nlp-1*. In glutamate-depleted mutants no increase is noticed, suggesting that NLP-1 acts as a co-transmitter for glutamate by decreasing its effect (Chalasani et al., 2010).

To identify the receptor through which NLP-1 is signaling, Chalasani et al. (2010) looked for orphan GPCRs expressed in neurons that are connected to the AWC sensory neurons. A knockout mutation of NPR-11, resulted in a similar phenotype as displayed by the *nlp-1* mutant. A calcium based assay confirmed the NLP-1/NPR-1 interaction.

**Figure 5:**
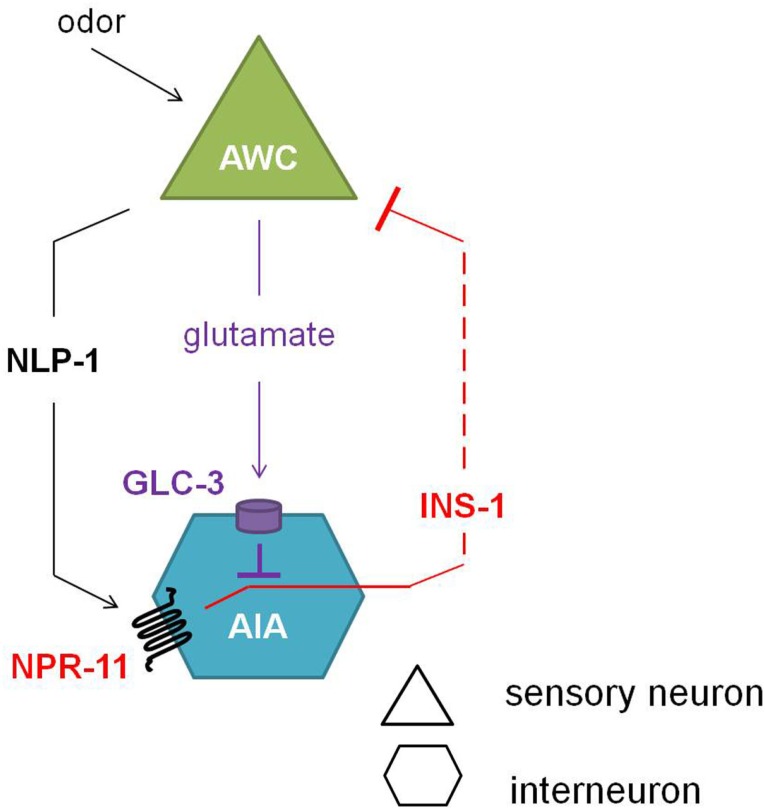
**Neuropeptide feedback regulation of the AWC sensory neurons**. The AIA interneurons are inhibited via the glutamate-gated chloride channel GLC-3 upon release of the neurotransmitter glutamate from the AWC neurons. Alternatively, when odor is sensed, the AWC neurons release NLP-1, which in turn activates NPR-11 on the AIA interneurons. Upon activation of NPR-11, INS-1 is released, inhibiting AWC activity and thereby reducing its inhibition on AIA (adapted from Chalasani et al., 2010).

Comparison of the calcium response of AWC neurons during the local search behavior upon food removal suggested that NPR-11 activation by NLP-1 evokes a negative feedback loop which dampens AWC activity (Figure 5). NPR-1 is expressed in the AIA interneurons which also express the insulin-like peptide INS-1. Indeed, an *ins-1* mutant shows the same increase in turning rates upon food removal as the *nlp-1* and *npr-11* mutants. Calcium imaging of the AWC neurons could confirm the role of INS-1 as a suppressor of AWC activity (Chalasani et al., 2010).

### Conservation of GnRH signaling

Gonadotropin releasing hormone is mainly known for its role in reproduction in vertebrates (Kah et al., 2007). The GnRH receptor and its ligand are highly conserved in vertebrates and homologs of the receptor are predicted in a variety of invertebrates (Roch et al., 2011). Remarkably, insect GnRH receptor orthologs are activated by adipokinetic hormone (AKH), corazonin, and AKH/corazonin-related peptide (ACP), which are known to be involved in energy metabolism, pigmentation, and cardiac regulation (Park et al., 2002; Staubli et al., 2002; Hansen et al., 2010; Lindemans et al., 2011; Roch et al., 2011). The genome of *C. elegans* is predicted to encode for a family of eight GnRH-related receptor genes (*gnrr-1* to *gnrr-8*). Only one of these receptors (GNRR-1, isoform a) has been deorphanized (Lindemans et al., 2009a). Since *Drosophila melanogaster* AKH (*Dm-*AKH) was capable of activating this receptor, the authors performed an *in silico* search for an AKH-GnRH-like peptide in *C. elegans*. This way, they were able to identify the decapeptide NLP-47 (pQMTFTDQWT) as the endogenous ligand for GNRR-1a (EC_50_ = 150 nM; Lindemans et al., 2009a). AKH is known to regulate lipid mobilization during flight in insects (Gäde and Auerwald, 2003). Fat contents were examined by performing an RNAi knockdown of *gnrr-1* and/or *nlp-47*. Unfortunately, no significant differences between knockdowns and wild type were observed (Lindemans et al., 2009a). Nevertheless, injection of synthetic *Ce*-AKH-GnRH into the cockroach *Periplaneta americana* resulted in a significant increase in the levels of hemolymph carbohydrates. A delay in egg-laying could be observed after both *gnrr-1* and *nlp-47* knockdown (Lindemans et al., 2009a). The identification of an AKH-GNRH-like signaling system involved in reproduction is an interesting finding and could be a key to the interplay between reproduction and energy metabolism.

Since no clear ortholog for GnRH was found in insects and nematodes, it was proposed that GnRH has been preserved in lophotrochozoans, but lost in the ecdysozoans (Tsai and Zhang, 2008). Nevertheless, phylogenetic analysis of the ligands of the ecdysozoan GnRH receptors suggests that AKH and corazonin share a common ancestor with GnRH (Lindemans et al., 2011; Roch et al., 2011).

### The NMU-like signaling pathway

In vertebrates, NMU is a highly conserved neuropeptide that plays a fundamental role in key physiological processes such as smooth muscle contraction, regulation of blood pressure, feeding and energy homeostasis, stress responses, and immune regulation (Brighton et al., 2004). All NMU peptides isolated in vertebrates have an identical C-terminal pentapeptide (FRPRNamide; Brighton et al., 2004). The presence of an NMU-like receptor in invertebrates was first reported for the fruitfly *D. melanogaster*. The fruitfly genome encodes four NMU receptor homologs. These receptors are activated by pyrokinin neuropeptides (PRXamide) and are involved in many functions such as feeding behavior and visceral muscle contraction (Schoofs et al., 1993; Park et al., 2002; Melcher and Pankratz, 2005).

The *C. elegans* genome encodes four NMU receptor homologs. To date, only NMUR-1 has an assigned phenotype. Wild type *C. elegans* display an altered lifespan depending on the type of food source they live on. In 2010, NMUR-1 was demonstrated to be involved in this food source dependent regulation of lifespan (Maier et al., 2010). So far, the activating ligand of NMUR-1 has not yet been identified. In contrast, though still a receptor of unknown function, NMUR-2 has recently been deorphanized based on an *in silico* search for *C. elegans* homologs of the *Drosophila* pyrokinin peptides. This revealed three putative PRXamide peptides, all encoded by the same peptide precursor gene *nlp-44*. Only one of these peptides, AFFYTPRI-NH2, could activate NMUR-2 (Lindemans et al., 2009b).

### PDF-like signaling: Locomotion and reproduction

In *C. elegans*, the G protein-coupled PDF receptors of the secretin receptor family PDFR-1a, b, c, d, and e represent five splice isoforms of *pdfr-1* (Janssen et al., 2008a; Barrios et al., 2012). Their endogenous neuropeptide ligands PDF-1a, PDF-1b, and PDF-2, encoded by *pdf-1* and *pdf-2*, are all of the NLP-type. All three PDF peptides are able to bind PDFR-1a, b and c; though with significant differences in affinity (Janssen et al., 2008a). PDFR-1a and PDFR-1b signaling occurs via a Gα_s_ type of G protein, while PDFR-1c signaling occurs through a Gα_i/o_ type of G protein (Janssen et al., 2009). PDFR-1d and e were only recently recovered from cDNA (Barrios et al., 2012), and have not yet been characterized in detail. The PDF-like neuropeptide pathway is highly conserved in nematodes, and PDF neuropeptides are also found in insects and crustaceans. In the latter, they were initially discovered and named pigment dispersing hormones (PDHs; Rao and Riehm, 1993; Janssen et al., 2009; Meelkop et al., 2011; Temmerman et al., 2011). Furthermore, all three *C. elegans* PDF receptors are closely related to insect orthologs, such as the *D. melanogaster* PDF receptor, and are distantly related to the vertebrate calcitonin GPCRs and vasoactive intestinal peptide (VIP) receptors (Janssen et al., 2008a). In *C. elegans*, the *pdfr-1* gene is expressed in every body wall muscle cell and, like *pdf-1* and *pdf-2*, in neuronal cells that are involved in the sensing and integration of environmental stimuli and the control of locomotion (Janssen et al., 2008a, 2009).

So far, functional characterization reveals that the PDF signaling system of *C. elegans* is involved in both locomotion and egg-laying, which stresses the pleiotropic nature of its biological functions. The *pdf-1(tm1996)* loss-of-function mutant shows locomotion defects by moving slower and executing more reversals than wild type worms. This locomotion phenotype is recapitulated by the overexpression of PDF-2 (Janssen et al., 2008a). *pdf-2 (tm4393)* deletion mutants conduct fewer backward/forward transitions than wild type animals, suggesting that PDF-1 and PDF-2 neuropeptides exert antagonistic effects on locomotion via PDFR-1. Furthermore, the PDF receptor loss-of-function mutant *pdfr-1(lst34)* turned out to have similar locomotion defects as the *pdf-1(tm1996)* mutant. Three splice variants of *pdfr-1* (a, b, c) were proven to be involved in the regulation of locomotion (Meelkop et al., 2012). The PDF system is also implicated in reproduction, as the timing of egg-laying appears to be delayed in *C. elegans* *pdf-1(tm1996)*, *pdf-2(tm4393)*, and *pdf-2(tm4780)* deletion mutants (Meelkop et al., 2012). The b and d isoforms could rescue a male-specific defect in mate-searching behavior. This defect is mediated through PDF-1 peptides, but not PDF-2; and seems to be needed in gender-shared neurons for the regulation of this sex-specific behavior (Barrios et al., 2012). Functions for PDF signaling in locomotion and reproduction have been demonstrated in other invertebrate species as well (Renn et al., 1999; Helfrich-Forster et al., 2000; Hamanaka et al., 2005). Recently, proteomic analysis proposed the involvement of PDF signaling in lipid metabolism and stress resistance (Temmerman et al., 2012).

### The CCK/gastrin-like signaling system: Food metabolism

Cholecystokinin and gastrin are well-characterized peptide hormones in vertebrates. By acting on two conserved GPCRs, CCK1R, and CCK2R; they are implicated in a variety of digestive functions including the stimulation of digestive enzyme production, intestinal motility, and the promotion of satiety in order to regulate food intake (Konturek et al., 2003; Dufresne et al., 2006; Clerc et al., 2007). In arthropods, the sulfakinin (SK) family of neuropeptides is both structurally and functionally related to the well-conserved vertebrate CCK and gastrin peptides (Schoofs and Nachman, 2006). A Basic Local Alignment Search Tool (BLAST) analysis of the *C*. *elegans* genome revealed *ckr-1* and *ckr-2* as the homologous genes of the vertebrate CCK/gastrin receptors and their SK counterparts in insects (Kubiak et al., 2002; Meeusen et al., 2003; McKay et al., 2007). The *ckr-2* gene encodes two splice isoform receptors, CKR-2a and CKR-2b, which belong to the rhodopsin GPCR family. By use of a reverse pharmacology approach, the endogenous *C. elegans* NLP-12a and NLP-12b neuropeptides – encoded by the *nlp-12* gene – were appointed the CCK/gastrin-like ligands of CKR-2a and CKR-2b (Janssen et al., 2008b). Signaling of the CCK receptors occurs through a Gα_q_ type of G protein. The *nlp-12* gene is expressed in a single tail neuron, identified as DVA, while *ckr-2* is expressed in cholinergic and GABAergic motor neurons (Janssen et al., 2008b; Hu et al., 2011).

**Figure 6:**
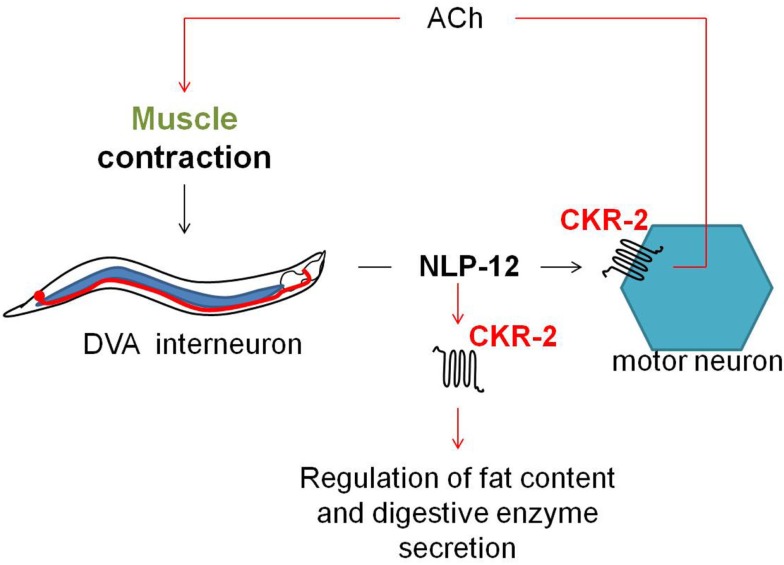
**Upon muscle contraction, NLP-12 neuropeptides are released by a single tail neuron, DVA**. Subsequent activation of the NLP-12 receptor, CKR-2, potentiates transmission at cholinergic neuromuscular junctions, thereby providing a mechanism for proprioceptive control of locomotion (Hu et al., 2011). NLP-12 signaling through CKR-2 also appears to be involved in the regulation of fat storage and digestive enzyme production (Janssen et al., 2008b).

The *C. elegans* *ckr-2(tm3082)* receptor mutant displays decreased intestinal amylase activity/secretion relative to wild type worms, suggesting the involvement of CKR-2 signaling in the stimulation of digestive enzyme secretion. The CCK/gastrin signaling system also appears to be involved in the control of fat storage since *ckr-2(tm3082)* as well as *nlp-12(ok335)* deletion mutants show an increased fat content compared to wild type animals (Janssen et al., 2008b). Both observations are in accordance with the functions attributed to the CCK/gastrin signaling system in vertebrates and the SK signaling system in arthropods. Recently, Hu et al. (2011) suggested a mechanosensory feedback loop (Figure 6) for proprioceptive control of normal locomotion, whereby muscle contraction aids the secretion of NLP-12 by the stretch-activated DVA neuron. Subsequent signaling of NLP-12 through CKR-2 enhances presynaptic ACh release to potentiate transmission at neuromuscular junctions and as such adjust the pattern of locomotion. Correspondingly, a significantly reduced locomotion rate was observed for both the *ckr-2(tm3082)* and *nlp-12(ok335)* mutants compared to wild type worm.

### An FaRP signaling pathway involved in egg-laying behavior

**Figure 7:**
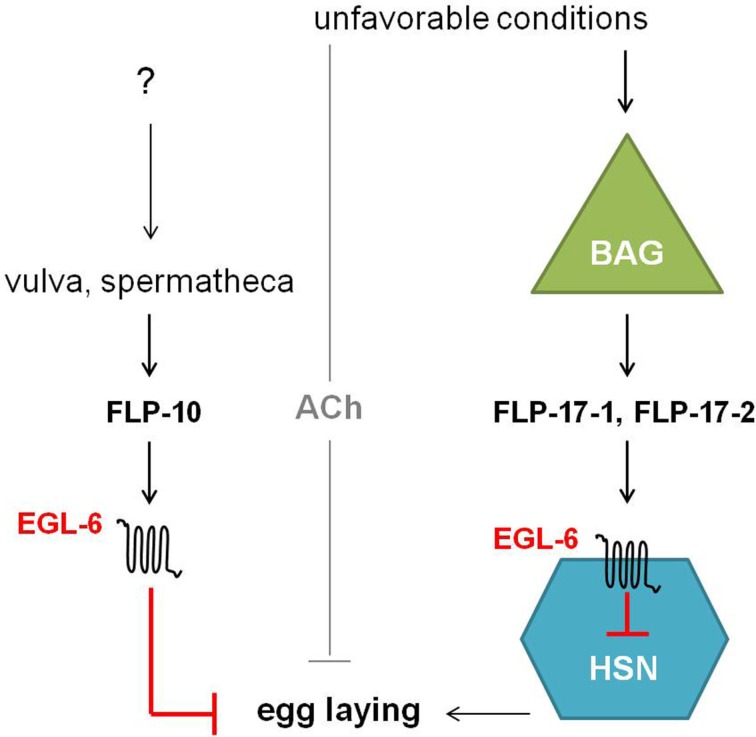
**Unfavorable conditions cause the release of FLP-17 neuropeptides from BAG neurons**. These neurohormones activate EGL-6 in HSN neurons in order to inhibit egg-laying. Release of FLP-10 by the vulva and spermatheca along with subsequent signaling via EGL-6 also inhibits egg-laying. How parts of the hermaphrodite’s reproductive system might inhibit egg-laying is not yet fully understood. In parallel to peptidergic inhibition, cholinergic signals inhibit egg-laying upon unfavorable conditions (Ringstad and Horvitz, 2008).

The *egl-6* gene encodes two GPCR isoforms that are both involved in the inhibition of egg-laying. In comparison with wild type, *egl-6(n592)* and *egl-6* overexpression mutants display slower egg-laying rates and longer retention of embryos. Allele *n592* appeared to be a gain-of-function mutation in *egl-6*, increasing its inhibiting activity (Ringstad and Horvitz, 2008). Similar to NPR-11, ligands for EGL-6 were first suggested by looking at neuropeptides displaying the defective egg-laying phenotype of *egl-6* overexpression when they are overexpressed in wild type worms but not in *egl-6* deletion mutants. This way, *flp-10* and *flp-17* turned out to encode for the ligands of EGL-6. In addition, a *Xenopus laevis* oocyte assay demonstrated that FLP-10, FLP-17-1, and FLP-17-2 were able to unambiguously activate the EGL-6 GPCR at nanomolar concentrations (Ringstad and Horvitz, 2008). These FaRPs signal from multiple cell types via EGL-6 in a Gα_i/o_-dependent manner to inhibit egg-laying (Figure 7). In response to environmental cues, FLP-17 neurohormones are principally expressed in BAG sensory neurons and thought to modulate egg-laying behavior by acting on EGL-6 in HSN motor neurons. The latter neurons are known to stimulate the action of vulval muscles and are involved in egg-laying (Trent et al., 1983; White et al., 1986). The non-neuronal expression of FLP-10 peptides in parts of the hermaphrodite’s reproductive system also inhibits egg-laying (Kim and Li, 2004; Ringstad and Horvitz, 2008). Upon unfavorable conditions, signaling through ACh also inhibits egg-laying in parallel to the aforementioned peptidergic inhibition (Ringstad and Horvitz, 2008).

### VP/OT signaling: Gustatory associative learning and reproduction

Recently, a VP/OT-related signaling system has been identified in *C. elegans*. In mammals, this system is involved in a plethora of peripheral hormonal functions including water homeostasis, reproduction, and stress responses (van Kesteren et al., 1995; Aikins et al., 2008). These neuropeptides also function as neuromodulators in the central nervous system influencing social cognition and behavior, memory and learning (de Wied et al., 1993; Young and Wang, 2004; Meyer-Lindenberg et al., 2011). In the roundworm, a single VP/OT-like peptide, named nematocin (NTC-1), and two nematocin receptors (NTR-1 and NTR-2) are identified. The NTR-1 receptor is activated by the nematocin peptide in a dose-dependent way. On the other hand, the NTR-2 receptor is not directly activated by NTC-1 but co-expression of NTR-1 and NTR-2 is suggested to affect the intracellular levels of cAMP upon nematocin binding (Beets et al., 2012; Garrison et al., 2012).

In hermaphrodites, the *ntc-1* gene is mainly expressed in the DVA and AVK neurons. Since *ntr-1* is expressed in the left ASE (ASEL) gustatory neurons, the ASH and ADF chemosensory neurons, which function in chemotaxis toward water-soluble cues, Beets et al. (2012) studied the salt chemotaxis behavior of *ntc-1* and *ntr-1* mutants. Similar to wild type worms, *ntc-1* and *ntr-1* mutants are attracted to low NaCl concentrations. When pre-exposed to these low NaCl concentrations in the absence of food, wild type worms show reduced attraction to or avoidance of NaCl, a behavioral switch termed gustatory plasticity (Hukema et al., 2008). However, the aversive response of pre-exposed worms is reduced in *ntc-1* and *ntr-1* mutants. These results indicate that nematocin signaling is implicated in gustatory associative learning, similar to the effects of VP and OT on mammalian cognition. Moreover, AVK-specific expression of *ntc-1* and ASEL-specific expression of *ntr-1* in the *ntc-1* and *ntr-1* mutant background, respectively, partially restored gustatory plasticity. Genetic analysis and supplementation studies indicated that the TRPV channel protein OSM-9, the Gγ-subunit GPC-1 and serotonin and dopamine signaling interact with the nematocin pathway in regulating gustatory plasticity (Beets et al., 2012).

*ntc-1*, *ntr-1*, and *ntr-2* are expressed in sexually dimorphic patterns and have been shown to function in male mating behavior. *ntc-1, ntr-1*, and *ntr-2* mutant males perform poorly in several types of mating behaviors compared with wild type worms. Mutations in the NTR-1 and NTR-2 receptor cause partly overlapping defects in the mating response. Remarkably, cell-specific knockout of nematocin in the mechanosensory DVA neuron, which is not male-specific, seems to be responsible for most of the male mating defects. These findings indicate that nematocin signaling is necessary to coordinate male mating behaviors (Garrison et al., 2012).

## Conclusion

Despite the simplicity of its nervous system, *C. elegans* displays complex behaviors with a high level of plasticity and striking similarities to the functioning of “higher” nervous systems. The completely defined anatomy and wiring of the worm’s nervous system, combined with a rapid life cycle and powerful molecular and genetic tools, have allowed the dissection of neuropeptidergic signaling networks with single cell resolution. As in other animals, neuropeptides in *C elegans* signal through GPCRs. Many predictions have been made about neuropeptide GPCR encoding genes in the *C. elegans* genome. This review brings the number of predicted neuropeptide GPCRs up to 128. High-throughput RNAi and mutagenesis studies of orphan neuropeptide GPCRs in *C. elegans* revealed their involvement in a broad repertoire of behaviors. However, cognate ligands for only 22 of the predicted neuropeptide GPCRs have been identified and only eight of these receptors are functionally characterized. These well-defined neuropeptidergic signaling systems play crucial roles in key physiological processes such as reproduction, locomotion, and lipid metabolism, as well as in social and foraging behaviors. The broad functioning of these neuropeptide GPCRs in nematode physiology emphasizes the pivotal role of neuropeptidergic signaling in *C. elegans*. The development of high-throughput deorphanization systems in combination with an advanced genetic toolbox will allow further functional characterization of neuropeptide GPCRs in *C elegan*s, likely increasing our understanding of peptidergic signaling systems in other organisms as well.

## Conflict of Interest Statement

The authors declare that the research was conducted in the absence of any commercial or financial relationships that could be construed as a potential conflict of interest.

## Appendix

**Table A1:** **List of sequences of neuropeptide ligands produced by neuropeptide precursors and sorted according to the neuropeptide *C. elegan**s* GPCRs they activate**.

Neuropeptide GPCR	Neuropeptide gene	Neuropeptide precursor gene	Neuropeptide sequence
NPR-1	*flp-18*	FLP-18-1	(DFD)GAMPGVLRFG
		FLP-18-2	EMPGVLRFG
		FLP-18-3	(SYFDEKK)SVPGVLRFG
		FLP-18-4	EIPGVLRFG
		FLP-18-5	SEVPGVLRFG
		FLP-18-6	DVPGVLRFG
	*flp-21*	FLP-21	GLGPRPLRFG
NPR-3	*flp-15*	FLP-15-1	GGPQGPLRFG
		FLP-15-2	RGPSGPLRFG
NPR-4	*flp-1*	FLP-1-6	(K)PNFLRFG
	*flp-4*	FLP-4-2	ASPSFIRFG
	*flp-18*	FLP-18-1	(DFD)GAMPGVLRFG
		FLP-18-2	EMPGVLRFG
		FLP-18-3	(SYFDEKK)SVPGVLRFG
		FLP-18-4	EIPGVLRFG
		FLP-18-5	SEVPGVLRFG
		FLP-18-6	DVPGVLRFG
NPR-5a	*flp-1*	FLP-1-2	SQPNFLRFG
	*flp-3*	FLP-3-1	SPLGTMRFG
		FLP-3-3	SAEPFGTMRFG
		FLP-3-6	EDGNAPFGTMRFG
		FLP-3-8	SADDSAPFGTMRFG
	*flp-18*	FLP-18-1	(DFD)GAMPGVLRFG
		FLP-18-2	EMPGVLRFG
		FLP-18-3	(SYFDEKK)SVPGVLRFG
		FLP-18-4	EIPGVLRFG
		FLP-18-5	SEVPGVLRFG
		FLP-18-6	DVPGVLRFG
	*flp-21*	FLP-21	GLGPRPLRFG
NPR-5b	*flp-1*	FLP-1-2	SQPNFLRFG
	*flp-3*	FLP-3-1	SPLGTMRFG
		FLP-3-3	SAEPFGTMRFG
		FLP-3-6	EDGNAPFGTMRFG
		FLP-3-8	SADDSAPFGTMRFG
	*flp-4*	FLP-4-2	ASPSFIRFG
	*flp-18*	FLP-18-1	(DFD)GAMPGVLRFG
		FLP-18-2	EMPGVLRFG
		FLP-18-3	(SYFDEKK)SVPGVLRFG
		FLP-18-4	EIPGVLRFG
		FLP-18-5	SEVPGVLRFG
		FLP-18-6	DVPGVLRFG
	*flp-21*	FLP-21	GLGPRPLRFG
NPR-6	*flp-18*	FLP-18-3	(SYFDEKK)SVPGVLRFG
		FLP-18-6	DVPGVLRFG
	*flp-21*	FLP-21	GLGPRPLRFG
NPR-10a	*flp-3*	FLP-3-1	SPLGTMRFG
		FLP-3-3	SAEPFGTMRFG
		FLP-3-5	ASEDALFGTMRFG
		FLP-3-6	EDGNAPFGTMRFG
		FLP-3-7	EAEEPLGTMRFG
		FLP-3-8	SADDSAPFGTMRFG
NPR-10b	*flp-18*	FLP-18-1	(DFD)GAMPGVLRFG
		FLP-18-3	(SYFDEKK)SVPGVLRFG
		FLP-18-4	EIPGVLRFG
		FLP-18-5	SEVPGVLRFG
		FLP-18-6	DVPGVLRFG
NPR-11	*flp-1*	FLP-1-6	(K)PNFLRFG
	*flp-5*	FLP-5-2	AGAKFIRFG
	*flp-14*	FLP-14	KHEYLRFG
	*flp-18*	FLP-18-3	(SYFDEKK)SVPGVLRFG
	*flp-21*	FLP-21	GLGPRPLRFG
	*nlp-1*		MDANAFRMSFG
FRPR-3	*flp-7*	FLP-7-1	TPMQRSSMVRFG
		FLP-7-2	SPMQRSSMVRFG
		FLP-7-3	SPMERSAMVRFG
	*flp-11*	FLP-11-1	AMRNALVRFG
FRPR-18a	*flp-2*	FLP-2-1	LRGEPIRFG
FRPR-18b		FLP-2-2	SPREPIRFG
NPR-22a	*flp-1*	FLP-1-6	(K)PNFLRFG
	*flp-7*	FLP-7-1	TPMQRSSMVRFG
		FLP-7-2	SPMQRSSMVRFG
		FLP-7-3	SPMERSAMVRFG
		FLP-7-4	SPMDRSKMVRFG
	*flp-9*	FLP-9	KPSFVRFG
	*flp-11*	FLP-11-1	AMRNALVRFG
		FLP-11-2	ASGGMRNALVRFG
		FLP-11-3	NGAPQPFVRFG
	*flp-13*	FLP-13-4	AADGAPLIRFG
	*flp-22*	FLP-22	SPSAKWMRFG
CKR-2a	*flp-1*	FLP-1-1	SADPNFLRFG
	*nlp-12*		DYRPLQFG
			DGYRPLQFG
CKR-2b	*nlp-13*		SSSMYDRDIMSFG
	*nlp-14*		ALDGLDGSGFGFD
GNRR-1a	*nlp-47*	NLP-47	pQMTFTDQWT
NTR-1	*ntc-1*	NTC-1	CFLNSCPYRRYG
NMUR-2	*nlp-44*		AFFYTPRIG
EGL-6a	*flp-10*	FLP-10	QPKARSGYIRFG
EGL-6b	*flp-17*	FLP-17-1	KSAFVRFG
		FLP-17-2	KSQYIRFG
PDFR-1a	*pdf-1*	PDF-1a	SNAELINGLIGMDLGKLSAVG
PDFR-1b		PDF-1b	SNAELINGLLSMNLNKLSGAG
